# Water polo coaches believe they gain an advantage by calling time-out before playing power-play, but is that really true?

**DOI:** 10.3389/fpsyg.2025.1548905

**Published:** 2025-02-19

**Authors:** Corrado Lupo, Damiano Li Volsi, Paolo Riccardo Brustio, Alexandru Nicolae Ungureanu

**Affiliations:** ^1^NeuroMuscularFunction Research Group, School of Exercise & Sport Sciences, University of Turin, Turin, Italy; ^2^Department of Medical Sciences, University of Turin, Turin, Italy; ^3^Department of Clinical and Biological Sciences, University of Turin, Turin, Italy; ^4^Department of Life Sciences and Systems Biology, University of Turin, Turin, Italy

**Keywords:** technical indicators, tactical indicators, margin of victory, time-out, power-play

## Abstract

**Introduction:**

The present study aimed to evaluate the impact of time-out on power-play outcomes both in elite senior and youth matches and in relation to final (margin of victory, MoV) and current (margin of advantage, MoA) match scores (i.e., winning in unbalanced games, MW; winning-draw-losing in close games, W-D-L; losing in unbalanced games, ML).

**Materials and methods:**

A total of 97 (seniors, *n* = 50; youth, *n* = 47) European Championship matches were analyzed, comparing power-plays preceded or not by a time-out in relation to the following offensive indicators: goal, exclusion, penalty, and no-goal.

**Results:**

The results reported that both senior and youth levels have been characterized by better power-play outcomes without time-out (higher goals scored: senior, *p* ≤ 0.01, youth, *p* ≤ 0.001; and lower “no goal” events: *p* ≤ 0.01, youth, *p* ≤ 0.01). Similar trends were observed with respect to the MoV. Specifically, in senior close games, there were both significantly higher goals scored (*p* ≤ 0.05) and fewer ‘no goal’ events (*p* ≤ 0.05), and these patterns were also evident among youth losing teams in unbalanced games. Differently, for MoA, both higher goals scored (*p* ≤ 0.01) and lower “no goal” events (*p* ≤ 0.01) emerged for senior losing teams in unbalanced games and youth close games (higher goals scored, *p* ≤ 0.01; and lower “no goal” events, *p* ≤ 0.05).

**Discussion:**

Therefore, the present study demonstrated that time-out tends to limit the success of the following power-play action and that MoV and MoA approaches do not overlap. As a consequence, coaches could benefit from these findings by being more aware of the actual time-out consequences on the following power-play as well as their defensive potentialities when the opponents call time-out.

## 1 Introduction

Water polo is considered a body-contact team sport characterized by technical and tactical strategic skills, high-speed swimming, shooting, and fighting to maintain or regain possession (Alcaraz et al., 2012). It is one of the oldest sports of the modern Olympic Games and is traditionally played as an aquatic team sport between two teams of 7 players each (including a goalkeeper), aiming to score more goals than the opposing team during the four 8-min game periods, with ball possessions limited to 30 s.

Starting in 2018, Féderation Internationale de Natation Amateur (FINA) experimented with new rules to create a faster and more dynamic game. These changes allowed goalkeepers to move beyond the half-distance line and touch the ball or players to shoot directly or swim and shoot without passing to another player after taking a corner throw [Federation Internationale de Natation (FINA), 2018]. Moreover, in case of a second possession in the same sequence, corner, or rebound after a shot or exclusion (with less than 20 s to the end of ball possession), a team has a maximum of 20 s for a new ball possession [Federation Internationale de Natation (FINA), 2021]. In addition, the penalty fault may occur if a defending player impedes an attacking player from behind within the 6-meter area when the attacking player is facing the goal and in the act of shooting unless the defending player makes contact only with the ball. Notably, according to new water polo rules, the penalty is allowed for this specific playing phase even if the offensive player holds the ball in their hand.

In terms of research, technical and tactical aspects of water polo have been substantially investigated with particular reference to different competition levels (Lupo et al., 2010), sex (Lupo et al., 2011; Menescardi et al., 2018; Mirvic et al., 2019), age categories (Lupo et al., 2009; Ruano et al., 2016), playing (Canossa et al., 2022) and coaching (Perazzetti et al., 2023b; Barrenetxea-Garcia et al., 2024) behaviors, performance evaluation (Perazzetti et al., 2023a). A particular emphasis has also been provided on score differences at the beginning of each quarter (Marcelino et al., 2012; Gómez et al., 2014; Ruano et al., 2016). Similarly, notational analyses have been provided, considering the ending score of the match (i.e., the margin of victory, MoV), for technical and tactical profiles related to winning and losing teams performing close (i.e., 1–3 goal difference at the end of the match) and unbalanced (i.e., more than 3 goal of difference) games, both in men’s (Lupo et al., 2012) and women’s (Lupo et al., 2014) world championships. Nevertheless, no information has been provided in considering the analysis of each playing action according to the current goal differences between teams (i.e., margin of advantage, MoA), which could contribute to a better understanding of the real playing teams’ requests and possibilities during a water polo match.

In addition to technical and tactical aspects, recent developments in international water polo rules have also addressed events that influence these factors, such as time-outs. In fact, after a few experimental seasons (2013/14–2018/19) in which each water polo team could request one time-out for each game period, the rules were revised to reinstate the previous regulation. Teams are now permitted to request only two time-outs per match, regardless of the game period [Federation Internationale de Natation (FINA), 2024].

A systematic investigation of the effects of calling a time-out has primarily focused on teams from the Adriatic Water Polo League (Hraste et al., 2020). Findings indicated similar goal frequencies (i.e., 41.6% for power-play actions following a time-out and 43.2% without a time-out), although these conclusions were based on descriptive statistics. These contrasts with earlier water polo studies reported significant differences because power-plays (i.e., playing phase played following an exclusion foul of a defensive player who has to go out of the court for 20 s) resulted more successful without a time-out (44.7%) compared to those preceded by a time-out (31.25%) (Platanou, 2008). In the study by Hraste et al. (2020), teams were considered according to three team-level sub-groups, reporting effects between each team group interaction for goals scored during power-plays performed without time-out. However, both abovementioned studies referred to past water polo rules where teams could benefit from one time-out a quarter (Platanou, 2008) and the 20-s power-play situation was played within a 30-s action period (Platanou, 2008; Hraste et al., 2020). In contrast, another study (Saavedra et al., 2020) examined the current water polo rules regarding time-outs and power-play periods but focused only on comparing time-out frequency between winning and losing teams without evaluating their impact on subsequent actions.

Therefore, the literature still lacks a reference to clarify whether the coaching decision to call a time-out before performing a power-play represents an advantage (e.g., to better organize the consequent offensive action) for a team competing at an elite level according to the current water polo rules. As a consequence, an analysis focused on time-out effects, considering different ages, competition levels, and margins of victory and advantage, could substantially improve the water polo coaches’ and sports scientists’ awareness of the real consequences of this particular playing event.

For these reasons, this study aimed to investigate technical and tactical factors of men’s water polo by evaluating the impact of calling a time-out on power-play outcomes (i.e., goal, exclusion, penalty, and no-goal) both in elite senior and youth matches (i.e., European Championships) and in relation to the classification of power-play according to different final (MoV) and current (MoA) match scoring (i.e., winning in unbalanced games, MW; winning-draw-losing in close games, W-D-L; losing in unbalanced games, ML).

## 2 Materials and methods

### 2.1 Participants

A notational analysis was performed on 97 men’s European water polo championship matches. In particular, the sample of matches consists of the following: 17 from the 2020 Senior Men’s European Water Polo Championship (Budapest, Hungary; January 14–26; 11 qualifications; 2 quarter-finals; 4 semi-finals, 2 for 5°–8° place, 1 for 9°–12° place, 1 for 1°–4° place; for a total of 14 teams); 33 from the 2022 Men’s European Water Polo Championship (Split, Croatia; August 29–September 10; 20 qualifications; 3 quarter-finals; 6 semi-finals, 2 for 1°–4° place, 1 for 5°–8° place, 3 for 9°–12° place; 4 finals, 1 for 1°–2° place, 1 for 3°–4° place, 1 for 5°–6° place, 1 for 7°–8° place; 16 teams); and 47 from the 2023 Men’s European U17 Water Polo Championships (Manisa, Turkey, August 8–14; 27 qualifications; 4 quarter-finals; 8 semi-finals, 2 for 13°–16° place, 2 for 9°–12° place, 2 for 5°–8° place, 2 for 1°–4° place; 8 finals, 1 for 15°–16° place, 1 for 13°–14° place, 1 for 11°–12° place, 1 for 9°–10° place, 1 for 7°–8° place, 1 for 5°–6° place, 1 for 3°–4° place, 1 for 1°–2° place; 16 teams).

### 2.2 Procedures

All water polo full matches, both for the 2020 and 2022 Men’s European Water Polo Championship and 2023 Men’s European U17 Water Polo Championships, were downloaded online from the YouTube European Aquatics channel.^1^ A notational analysis was successfully performed using LongoMatch video analysis software v. 1.3.7. (Fluendo, Barcellona, Spain), and after creating a specific dashboard for collecting data.

The total number of time-outs was counted, specifically considering if it was called before a power-play action (i.e., after an opponent’s temporary exclusion) or during another situation (i.e., after a goal scored by the opponent or during a situation characterized by a number of offensive players relative to the ball position, never larger than that of the defense). In addition, the percentage distribution of goals, opponent’s exclusions, penalties, and other no goal outcomes (e.g., shot without goal, ball stolen by an opponent, shot clock violation, offensive fault, and off-side fault) were counted for each team performing power-plays (i.e., all offensive actions following of the temporary exclusion of an opponent defensive player, regardless of the teams’ arrangements) played with and without apreceding time-out. All analyzed actions were classified in relation to MoV and MoA categories, as well as senior and youth competition levels. In particular, for MoV, all power-plays performed by a team playing within a single match were considered according to the final score: winning teams in unbalanced games (i.e., with more than 3 goals), teams in close games (i.e., winning or losing with 1-2-3 goals or draw matches), and losing teams in unbalanced games (i.e., losing with more than 3 goals). Differently, for MoA, all power-plays performed by a team were classified according to its current score, considering the same goal threshold reported above for MoV. In line with previous notational analyses on water polo (Lupo et al., 2014, 2016), a single experienced analyst (who already experienced the notational analysis of more than 300 water polo games) analyzed two randomly selected matches to test either the intra- or inter-observer reliability. Three observers (i.e., the analyst of the study and two additional water polo coaches) scored the same two matches twice (i.e., observations separated by 7 days) to calculate the Lin’s concordance correlation coefficient (CCC) for each variable, and values of the same observer (CCC range = 0.94–1.00) and of all three observers (CCC range = 0.94–1.00), thus reporting the correspondent intra- and inter-observer reliabilities, respectively.

### 2.3 Data analysis

For each technical and tactical indicator, percentage mean values, standard deviations, and ranges of the power-play action outcomes were calculated for MoV and MoA categories and senior and youth competition levels.

Due to the investigated variables violating the normal distribution (assessed using the Shapiro-Wilk normality test), all data were analyzed using a non-parametric approach. Thus, the Mann–Whitney test was applied to compare power-plays with and without the previous call of a time-out. Finally, for each emerging difference, the phi effect sizes (ESs) were applied, considering 0.1, 0.3, and 0.5 as small, medium, and large ESs, respectively (Huck et al., 2004). Statistical analyses were applied using the IBM SPSS Statistics package (version 29, IBM Corp., New York, NY, USA), and the criterion for significance was set at a *p*-value of ≤0.05.

## 3 Results

On average, time-outs have been called 1.51 (33% in even action and 67% in power-plays) and 1.52 (40% in even action and 60% in power-plays) times a match from a single team in senior and youth matches. A total of 2041 (senior: 1059, in which 102, 10%, after time-out; youth: 982, in which 95, 10%, after time-out) power-plays were analyzed in the present study. In particular, for senior and MoV classification, 299 (*n* = 21, 7%, with time-out), 517 (*n* = 52, 10%, with time-out), and 231 (*n* = 28, 12%, with time-out) power-plays were analyzed for winning teams in unbalanced games, teams in close games, and losing in unbalanced games, respectively, whereas for senior and MoA classification, 164 (*n* = 12, 7%, with time-out), 731 (*n* = 63, 9%, with time-out), and 153 (*n* = 27, 18%, with time-out) power-plays were analyzed for winning teams in unbalanced games, teams in close games, and losing in unbalanced games, respectively. For youth and MoV classification, 228 (*n* = 22, 10%, with time-out), 491 (*n* = 45, 9%, with time-out), and 263 (*n* = 28, 11%, with time-out) power-plays were analyzed for winning teams in unbalanced games, teams in close games, and losing in unbalanced games, respectively, whereas, for youth and MoA classification, 92 (*n* = 15, 16%, with time-out), 754 (*n* = 61, 8%, with time-out), and 136 (*n* = 19, 14%, with time-out) power-plays were analyzed for winning teams in unbalanced games, teams in close games, and losing in unbalanced games, respectively.

Descriptive statistics (i.e., means, standard deviations, and ranges) and differences with corresponding effect size (ES) values of power-play outcomes, in relation to a playing situation preceded or not by a time-out, in senior and youth competition levels are reported in Table 1.

**Table 1:** Means, standard deviations, ranges, differences (i.e., *p* ≤ 0.05), and effect size (ES) values of senior and youth, in relation to each power-play outcome with time-out requested (To) or not (No To).

	Senior		Youth	
Power-play outcome	To (%)	No To (%)	To (%)	No To (%)
Goal	35 ± 44.3 (0–100)** ES = 0.1	43 ± 20 (0–89)	30 ± 43.1 (0–100)*** ES = 0.1	39.3 ± 20.3 (0–100)
Exclusion	0.7 ± 5.8 (0–50)	1 ± 3.3 (0–15)	4.1 ± 19.9 (0–100)	0.3 ± 1.6 (0–11)
Penalty	0 ± 0 (0–0)*** ES = 0.3	1.9 ± 4.9 (0–25)	0.7 ± 5.8 (0–50)*** ES = 0.1	2.3 ± 5.6 (0–33.3)
No-goal	64.2 ± 45 (0–100)** ES = 0.1	54 ± 18.9 (11–100)	65.3 ± 44.6 (0–100)** ES = 0.1	58.1 ± 19.3 (0–100)

** (*p* ≤ 0.01), and *** (*p* ≤ 0.001) difference with respect to action without time-out requested.

The same descriptive and statistical values reported in Table 1 were also considered to show the MoV and MoA effects of senior (Table 2) and youth (Table 3) matches.

**Table 2:** Means, standard deviations, ranges, differences (i.e., *p* ≤ 0.05), and effect size (ES) values of margins of victory and advantage for senior, in relation to each power-play outcome with time-out requested (To) or not (No To).

	Margin of victory						Margin of advantage					
	Winning teams in unbalanced games (MW)		Teams in close games (W-D-L)		Losing teams in unbalanced games (ML)		Winning teams in unbalanced games (MW)		Teams in close games (W-D-L)		Losing teams in unbalanced games (ML)	
Power-play outcome	To	No To	To	No To	To	No To	To	No To	To	No To	To	No To
Goal (%)	55.9 ± 49.6 (0–100)	57.9 ± 16.4 (18–89)	32.4 ± 42.8 (0–100)* ES = 0.1	41.1 ± 17.2 (10–80)	22.5 ± 38 (0–100)	31.4 ± 19.4 (0–75)	58.3 ± 51.5 (0–100)	51.8 ± 31.9 (0–100)	33.7 ± 44.9 (0–100)	41.1 ± 35.4 (0–100)	17.5 ± 33.5 (0–100)** ES = 0.3	35.6 ± 25.4 (0–100)
Exclusion (%)	0 ± 0 (0–0)	0.3 ± 1.4 (0–7)	1.4 ± 8.2 (0–50)	1.2 ± 3.7 (0–14)	0 ± 0 (0–0)	1.3 ± 3.8 (0–15)	0 ± 0 (0–0)	0.6 ± 3.5 (0–20)	1 ± 7.1 (0–50)	0.7 ± 4.6 (0–50)	0 ± 0 (0–0)	1.9 ± 6.3 (0–25)
Penalty (%)	0 ± 0 (0–0)	1.4 ± 3.3 (0–10)	0 ± 0 (0–0)** ES = 0.3	2 ± 5.2 (0–25)	0 ± 0 (0–0)	2.3 ± 5.9 (0–20)	0 ± 0 (0–0)	0 ± 0 (0–0)	0 ± 0 (0–0)	1.9 ± 9.3 (0–100)	0 ± 0 (0–0)	2.1 ± 8.8 (0–50)
No-goal (%)	44.1 ± 49.6 (0–100)	40.4 ± 15.5 (11–75)	66.2 ± 44.2 (0–100)* ES = 0.2	55.6 ± 16.7 (20–90)	77.5 ± 38 (0–100)* ES = 0.2	65 ± 18.6 (25–100)	41.7 ± 51.5 (0–100)	47.7 ± 32.2 (0–100)	65.3 ± 45.9 (0–100)	57.4 ± 36.7 (0–100)	82.5 ± 33.5 (0–100)** ES = 0.4	60.4 ± 24 (0–100)

*(*p* ≤ 0.05), **(*p* ≤ 0.01) difference with respect to action without time-out requested.

**Table 3:** Means, standard deviations, ranges, differences (i.e., *p* ≤ 0.05), and effect size (ES) values of margins of victory and advantage for youth, in relation to each power-play outcome with time-out requested (To) or not (No To).

	Margin of victory						Margin of advantage					
	Winning teams in unbalanced games (MW)		Teams in close games (W-D-L)		Losing teams in unbalanced games (ML)		Winning teams in unbalanced games (MW)		Teams in close games (W-D-L)		Losing teams in unbalanced games (ML)	
Power-play outcome	To	No To	To	No To	To	No To	To	No To	To	No To	To	No To
Goal (%)	42.5 ± 49.4 (0–100)	44.2 ± 21.2 (11–100)	29.1 ± 42.8 (0–100)** ES = 0.2	41.7 ± 19.9 (0–80)	18.4 ± 34.2 (0–100)** ES = 0.2	29.7 ± 17.7 (0–67)	33.3 ± 48.8 (0–100)	41 ± 41.6 (0–100)	26.7 ± 44.2 (0–100)*** ES = 0.1	38.8 ± 35.2 (0–100)	40 ± 47.1 (0–100)	36.6 ± 37.5 (0–100)
Exclusion (%)	5 ± 22.4 (0–100)	0.5 ± 2.2 (0–11)	5.7 ± 23.5 (0–100)	0.2 ± 1.2 (0–8)	0 ± 0 (0–0)	0.3 ± 1.4 (0–7)	6.7 ± 25.8 (0–100)	0 ± 0 (0–0)	3.6 ± 18.9 (0–100)	0.2 ± 1.6 (0–17)	0 ± 0 (0–0)	0 ± 0 (0–0)
Penalty (%)	0 ± 0 (0–0)** ES = 0.4	4.2 ± 6.4 (0–20)	0 ± 0 (0–0)* ES = 0.2	1.5 ± 5.5 (0–33)	2.6 ± 11.5 (0–50)	1.8 ± 4.6 (0–17)	0 ± 0 (0–0)	4.8 ± 20.5 (0–100)	0 ± 0 (0–0)* ES = 0.1	2.3 ± 11.7 (0–100)	3.3 ± 12.9 (0–50)	2.9 ± 10.2 (0–50)
No-goal (%)	52.5 ± 49.9 (0–100)	51.2 ± 20.3 (0–86)	65.2 ± 45.3 (0–100)	56.7 ± 18.1 (20–100)	78.9 ± 34.6 (0–100)* ES = 0.2	68.3 ± 17.4 (33–100)	60 ± 50.7 (0–100)	54.3 ± 42.4 (0–100)	69.7 ± 45.9 (0–100)** ES = 0.1	58.7 ± 35.5 (0–100)	56.7 ± 45.8 (0–100)	60.6 ± 39.7 (0–100)

*(*p* ≤ 0.05), ** (*p* ≤ 0.01), and *** (*p* ≤ 0.001) difference with respect to action without time-out requested.

## 4 Discussion

To the best of our knowledge, this study is the first study systematically quantifying the impact of time-out on consequent power-play action in water polo games, by considering team technical and tactical indicators (i.e., goal, exclusion, penalty, and other outcomes), in elite men’s senior and youth matches, and in relation to different MoV and MoA categories.

As the main finding, the present study demonstrated how time-out tends to limit the success of scoring a goal during the following power-play action. Despite coaches usually calling time-outs aiming to organize the following offensive arrangement better and create the best solution to shot and score a goal (Platanou, 2008), the present study reported the opposite scenario in senior and youth elite men’s matches, even considering the two competition levels in relation to different MoV and MoA categories.

Singularly considering both senior and youth competition levels (i.e., the first aim of the study), it clearly emerged how goal scoring is more likely to occur without prior time-out than following one. Coherently, this trend is also strengthened by the “no goal” indicator, which groups negative power-play outcomes (e.g., shot without goal, ball stolen by an opponent, shot clock violation, offensive fault, and off-side fault), reporting a specular significance to the goal indicator. Therefore, these findings can be considered controversial to those of a previous study where no playing effects have been associated with time-outs in men’s Adriatic League matches (Hraste et al., 2020), even though different water polo rules were considered in these matches, with the possibility of calling a time-out during each quarter. Similarly, controversial results emerged for the women’s world championship (Ruiz-Lara et al., 2018), even though rules permitted only two time-outs for a team during the entire match. On the other hand, similar trends emerged for a less recent water polo competition (2006 European Championships, Budapest, Hungary), where power-plays without preceded time-outs determined a higher frequency of goals than after time-outs (Platanou, 2008).

Moreover, penalties (committed by the opponent’s defense), which can be considered as an extreme defensive playing attempt to prevent the opponents from scoring a goal, reported significantly higher values associated with the absence of a previous time-out. No effect emerged for exclusions obtained by offensive teams during power-plays with and without the presence of time-outs, either due to the reduced and episodical occurrence of this playing event in power-plays, often determining high standard deviation (e.g., power-plays in youth matches after time-out). Therefore, time-out seems more associable with a benefit for the defense, although this phase is paradoxically wanted by the coach gaining the ball possession, playing an offensive game phase.

For the second aim of this study, several differences emerged for MoV and MoA, both in senior and youth competition levels, tending to confirm the general finding for which no playing advantage is associated with the calling of a time-out. Nevertheless, different effects emerged for the two action classifications. For MoV in senior matches (Table 2), the results highlight how time-out is associated with lower goal scoring and higher “no goal” occurrences despite being significant only for close games (with small effect sizes). In addition, a better trend in power-plays without time-out is strengthened by the higher frequency (with a medium effect size) of penalties obtained. As a consequence, it is reasonable to speculate that the defense could get more advantage from the time-out period than the offensive team, effectively resting before the imminent inferiority playing situation and optimizing the corresponding arrangement, tending to confirm less recent water polo analysis (Platanou, 2008) and to diverge from the most recent ones (Ruiz-Lara et al., 2018; Hraste et al., 2020). Although unbalanced games reported similar time-out consequences to those of close games, a significant result emerged only for the “no goal” indicator in losing teams, probably because the limits of these teams in effectively benefitting from the superiority situation could be improved by a previous time-out, which could tend to advantage the defense instead of the offense teams as speculated above.

For MoA applied on senior matches (Table 2), significances (with medium effect sizes) emerged only for losing teams in unbalanced games, thus confirming the speculation for which a time-out is an advantage for defense. Differently, no effect was found for the other MoA categories. However, while close games reported values in line with the general tendency, a controversial scenario emerged for winning teams in unbalanced games, where the frequency of goals scored tends to be favored, such as “no goal” playing events seem to be minimized, by the presence of a time-out called previously to power-play. Although no effect emerged, these last results could suggest that the aim of effectively calling a time-out to provide beneficial feedback information for the team (Platanou, 2008) could be concrete only if the offensive team is winning with a high MoA. In other words, only in this scoring circumstance, coaches of the senior winning team seem to be able to effectively transfer their power-play tactical strategies to score a goal in the following action.

In youth competitions (Table 3), the general trend observed in senior matches is confirmed, indicating that time-outs provide a potential advantage to defensive rather than offensive teams. In particular, for MoV, effects both for goal and “no goal” indicators emerged only in losing teams in unbalanced games. These effects were more limited in the other two MoV categories. Specifically, in close games, only goals scored and penalties showed an advantage for power-plays without a time-out, while in unbalanced games, only penalties committed by the winning team benefited from the absence of a time-out. In addition, similarly for the senior level, no substantial time-out effects are associated with power-play outcomes for winning teams in unbalanced games. Therefore, MoV can add information on time-out effects on power-play outcome, which has been previously considered in general (Platanou, 2008) or in relation to quarters (Ruiz-Lara et al., 2018) and ranking of teams (Hraste et al., 2020).

However, youth matches classified in terms of MoA reported a scenario partially overlapping with respect to senior one. In fact, only power-plays in close games were affected by time-out, reporting advantages for the defense instead of the offense teams in terms of goal scored, penalties, and minor “no goal” events (with small effect sizes). In addition, although not statistically significant, losing teams executing power-plays in unbalanced youth games exhibited slightly higher goal-scoring rates and lower “no goal” rates, reversing the trend observed at the senior level.

In general, this study highlights how power-plays are differently affected in relation to different grades of MoV and MoA and how these two classifications determine divergent tendencies. This scenario is plausible because power-plays are recruited differently into MoV and MoA analyses despite referring to equal margins of goals. In particular, as remarked in a previous study (Lupo et al., 2014), a water polo match can be played with a fluctuating score for which a close or unbalanced MoV is decided only toward the end of the match. Therefore, in the present study, MoA was able to consider the exact score moment for teams, thus avoiding considering actions played in the same category during potentially different competitive pressures perceived by teams. However, this study has been mainly affected by the experimental limitation due to a reduced number of power-plays preceded by a time-out (i.e., not more than two cases a match according to rules), which have determined frequent high standard deviations and significances associated with low effect sizes. In addition, a potential and progressive adaptation of international water polo rule changes by referees from 2020 to 2023 may have provided a bias, particularly for identifying penalties, thus stimulating eventual changes in coaches’ strategies pertinent to time-outs. Finally, no analysis has been applied for specific power-play actions played without the interruption of a timeout for being highly advantaging situations (e.g., one offensive player against the opponent goal keeper, or primary counterattacks such as 2 vs. 1, or 3 vs. 2, etc.), and expected as more profitable than 6 (offensive) vs. 5 (defensive) players situations, usually played after a timeout. Conversely, also power-play originating during an offensive transition (i.e., swimming offensive phase toward opponent goal) has not specifically analyzed, despite they could expectable as less profitable than the 6 vs. 5 power-play (usually associated to timeout), being potentially characterized by late power-plays (with less than playing 20 seconds) with a complete offensive arrangement. Therefore, future research focused on the incidence of time-out on power-plays, even considering the above-mentioned specifications, or other power-play aspects (e.g., number of passes, assist side or shot side, etc.) as well as of the action before (leading to the temporary opponent exclusion), could provide further findings, especially whether new water polo rules will be applied.

## 5 Conclusion

The present study demonstrated how time-out tends to limit the success of scoring a goal during the following power-play action. Despite coaches usually calling time-out with the scope of better organizing the offensive strategies and improving the probability of scoring a goal, the present study reported the opposite trend, even with several significances in favor of a disadvantage for teams in offensive power-plays. Although this general scenario has also been confirmed for the specific MoV and MoA analyses, different effects emerged for these two approaches, demonstrating how they are not considerable as stackable. Hypothesizing that the MoA classification could provide more reliable data than MoV in consideration of the actual competitive pressure perceived by teams, coaches could consider avoiding calling time-out, especially when teams are largely losing, for senior level, and when teams are performing within a close MoA, for youth one, because they represent the specific moment in which time-out is highly harmful. On the other hand, coaches could benefit from this series of information to be more aware of their defensive potentialities in correspondence with a time-out called by the opponents.

## Data Availability

The raw data supporting the conclusions of this article will be made available by the authors without undue reservation.

## Footnotes

^1^ https://www.youtube.com/@EuropeanAquatics
