# Corrigendum: Water polo coaches believe they gain an advantage by calling time-out before playing power-play, but is that really true?

**DOI:** 10.3389/fpsyg.2025.1587001

**Published:** 2025-03-21

**Authors:** Corrado Lupo, Damiano Li Volsi, Paolo Riccardo Brustio, Alexandru Nicolae Ungureanu

**Affiliations:** ^1^NeuroMuscularFunction Research Group, School of Exercise & Sport Sciences, University of Turin, Turin, Italy; ^2^Department of Medical Sciences, University of Turin, Turin, Italy; ^3^Department of Clinical and Biological Sciences, University of Turin, Turin, Italy; ^4^Department of Life Sciences and Systems Biology, University of Turin, Turin, Italy

**Keywords:** technical indicators, tactical indicators, margin of victory, time-out, power-play

In the published article, there was an error.

A correction has been made to **Introduction**, paragraph 3. This sentence previously stated:

“playing behaviors (Canossa et al., 2022), performance evaluation (Perazzetti et al., 2023), and rule changes (Sahrom et al., 2018).”

The corrected sentence appears below:

“playing (Canossa et al., 2022) and coaching (Perazzetti et al., 2023b; Barrenetxea-Garcia et al., 2024) behaviors, performance evaluation (Perazzetti et al., 2023a).”

A correction has been made to **Materials and methods**, *Procedures*, paragraph 1. This sentence previously stated:

“A notational analysis was successfully performed using LongoMatch video analysis software v.1.3.7. (Fluendo, Barcellona, Spain).”

The corrected sentence appears below:

“A notational analysis was successfully performed using LongoMatch video analysis software v. 1.3.7. (Fluendo, Barcellona, Spain), and after creating a specific dashboard for collecting data.”

A correction has been made to **Materials and methods**, *Procedures*, paragraph 2. This sentence previously stated:

“In addition, the percentage distribution of goals, opponent's exclusions, penalties, and other no goal outcomes (e.g., shot without goal, ball stolen by an opponent, shot clock violation, offensive fault, and off-side fault) were counted for each team performing power-plays played with and without a preceding time-out.”

The corrected sentence appears below:

“In addition, the percentage distribution of goals, opponent's exclusions, penalties, and other no goal outcomes (e.g., shot without goal, ball stolen by an opponent, shot clock violation, offensive fault, and off-side fault) were counted for each team performing power-plays (i.e., all offensive actions following of the temporary exclusion of an opponent defensive player, regardless of the teams' arrangements) played with and without a preceding time-out.”

A correction has been made to **Materials and methods**, *Procedures*, paragraph 2. This sentence previously stated:

“to calculate ICC for each variable and values of the same observer (ICC range = 0.95–1.00) and of all three observers (ICC range = 0.95–1.00), thus reporting the correspondent intra- and inter-observer reliabilities, respectively.”

The corrected sentence appears below:

“to calculate the Lin's concordance correlation coefficient (CCC) for each variable, and values of the same observer (CCC range = 0.94–1.00) and of all three observers (CCC range = 0.94–1.00), thus reporting the correspondent intra- and inter-observer reliabilities, respectively.”

In the published article, there was an error.

A correction has been made to **Discussion**, paragraph 9. This sentence previously stated:

“Therefore, future research focused on the incidence of time-out on power-plays could provide further findings, especially whether new water polo rules will be applied.”

The corrected sentence appears below:

“Finally, no analysis has been applied for specific power-play actions played without the interruption of a timeout for being highly advantaging situations (e.g., one offensive player against the opponent goal keeper, or primary counterattacks such as 2 vs. 1, or 3 vs. 2, etc.), and expected as more profitable than 6 (offensive) vs. 5 (defensive) players situations, usually played after a time-out. Conversely, also power-play originating during an offensive transition (i.e., swimming offensive phase toward opponent goal) has not specifically analyzed, despite they could expectable as less profitable than the 6 vs. 5 power-play (usually associated to time-out), being potentially characterized by late power-plays (with less than playing 20 seconds) with a complete offensive arrangement. Therefore, future research focused on the incidence of time-out on power-plays, even considering the above-mentioned specifications, or other power-play aspects (e.g., number of passes, assist side or shot side, etc.) as well as of the action before (leading to the temporary opponent exclusion), could provide further findings, especially whether new water polo rules will be applied.”

In the published article, there was an error. Two citations were missed.

(Barrenetxea-Garcia et al., 2024; Perazzetti et al., 2023b) were not cited in the article. The citations have now been inserted in **Introduction**, line 63.

The corresponding references were added to the References section.

The authors apologize for these errors and state that this does not change the scientific conclusions of the article in any way. The original article has been updated.
